# Metal Content in *Valeriana officinalis* L. Root Commercialized in a Spanish Region (Tenerife, Canary Islands)

**DOI:** 10.3390/foods15050958

**Published:** 2026-03-09

**Authors:** Juan R. Jáudenes-Marrero, Ángel Gutiérrez-Fernández, Chaxiraxi Morales-Marrero, Carmen Rubio, Soraya Paz-Montelongo, Samuel Alejandro-Vega, Ramón A. Muñoz de Bustillo-Alfaro, Arturo Hardisson, Conrado Carrascosa, Susana Abdala Kuri, Adama Peña-Vera, Sandra Dévora-Gutiérrez, Daida Alberto-Armas

**Affiliations:** 1 Toxicology Area, Universidad de La Laguna, La Laguna, 38071 Santa Cruz de Tenerife, Spaincrubio@ull.edu.es (C.R.); salejand@ull.edu.es (S.A.-V.);; 2 Interuniversity Group of Environmental Toxicology, Food and Drug Safety, Universidad de La Laguna, La Laguna, 38071 Santa Cruz de Tenerife, Spain; 3 Area of Pharmacology, Universidad de la Laguna, La Laguna, 38071 Santa Cruz de Tenerife, Spainsabdala@ull.edu.es (S.A.K.); sdevora@ull.edu.es (S.D.-G.); 4 Department of Animal Pathology and Production, Bromatology and Food Technology, Faculty of Veterinary, University of Las Palmas de Gran Canaria, Arucas, 35413 Las Palmas, Spain

**Keywords:** valeriana, food supplements, risk assessment, toxic metals, valerian root

## Abstract

One of the most popular food supplements among the Canary population for the treatment of insomnia and mild anxiety is *Valeriana officinalis* L. (valerian), whose organ of use is the root. However, this plant is susceptible to the accumulation of certain metals, and a daily multi-dose treatment may be a dosage indication/regimen as multidose therapy. Therefore, there is an interest in determining its content of metals (Cd, Pb, Al, Cr, Cu, Li, Ni, Sr, Mo, Zn, Co, Fe, B, Mn, V, Ba, K, Na, Mg, Ca) to establish the possible toxicological risk of its consumption. The concentrations were determined by inductively coupled plasma optical emission spectrometry (ICP-OES) in a total of 23 samples (8 fragmented, 7 crushed, 8 pulverized). The Cd limit set by the European Pharmacopoeia (0.1 mg/kg) is more than doubled in six samples. The results showed that Pinisan (0.11 mg/kg), the EnRelax^®^ pill (0.12 mg/kg), the EnRelax^®^ infuser (0.13 mg/kg), Kneipp (0.15 mg/kg), Milvus (0.16 mg/kg) and one of the market samples (0.23 mg/kg) all exceed this parameter. However, the use of valerian root as a herbal plant or food supplement at therapeutic doses and in all studied dosages does not pose a toxicological risk based on the Estimated Daily Intake (EDI) of metals.

## 1. Introduction

In recent years, mental health problems have increased significantly. There are different factors that contribute to this, with the COVID-19 pandemic being one of the most recent [1]. Depression, stress and anxiety are prevalent pathologies that affect mental health [2] and often coexist associated with insomnia.

Insomnia is defined as a sleep disorder where there are difficulties in falling asleep and/or maintaining restful sleep [3], at least three times a week or for more than 3 months [4]. Insomnia can be sporadic or chronic and when it persists it is associated with functional and cognitive impairment with serious health consequences [4].

On the other hand, mild anxiety is a mental disorder characterized by feelings of excessive worry, nervousness, or fear that can interfere with daily life [5]. Common symptoms include restlessness, fatigue, difficulty concentrating, irritability, and sleep disturbances [6].

There are different treatments to control these pathologies: non-pharmacological treatments, pharmacological treatments, alternative medicine and cognitive-behavioral therapy [3]. Currently, the use of conventional medications to treat insomnia and mild anxiety has been the subject of controversy due to their toxicity profile; there are side effects and risk of dependence associated with drugs such as benzodiazepines [7]. For this reason, in recent years there has been a certain interest in safer alternatives such as traditional phytotherapeutic medicine.

**Figure 1:**
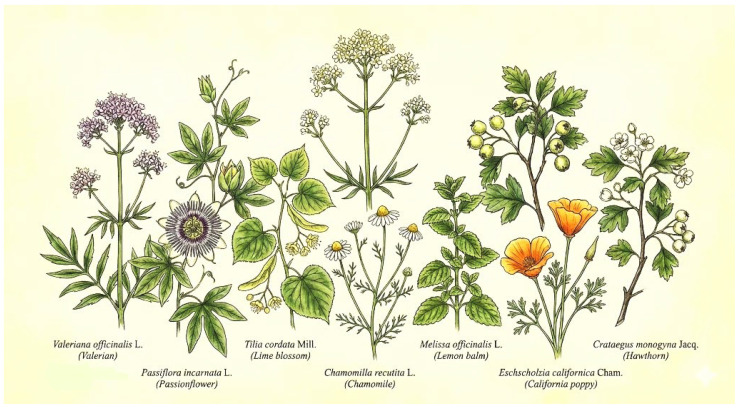
Different plants that are commonly used like valerian.

A large number of plants, used alone or in combination, are commonly used for the treatment of insomnia, including valerian (*Valeriana officinalis* L.), passionflower (*Passiflora incarnata* L.), lime blossom (*Tilia cordata* Mill.), chamomile (*Chamomilla recutita* L.), lemon balm (*Melissa officinalis* L.), California poppy (*Eschscholzia californica* Cham.) and hawthorn (*Crataegus monogyna* Jacq.) [8,9,10,11]. (Figure 1)

Ascanio-Umpiérrez [12] analyzed the consumption of medicinal plants indicated for insomnia and anxiety on the island of Tenerife and concluded that valerian, linden and passionflower were the most widely used medicinal plants [12]. Subsequently, Jensen et al. [2] carried out a review of medicinal plants used for mental health problems in the Mediterranean region, and among them valerian, lemon balm, and chamomile for their hypnotic activity stood out. Both authors agree that valerian is the most widely used medicinal plant.

**Figure 2:**
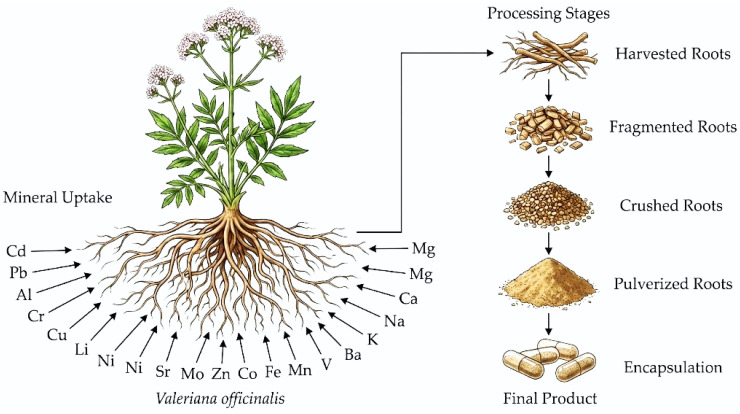
*Valeriana officinalis* and the valerian marketed root forms.

The rhizome, roots, and stolons of valerian are the plant organs that contain the active molecules. These organs are often referred to as “valerian root” [13] (Figure 2). Valoptriates and valerenic acid are the active ingredients responsible for the anxiolytic and sedative effect [14] of valerian.

Although the pharmacological activity of valerian is not completely defined. Some evidence establishes that it begins with the inhibition of γ-aminobutyric acid (GABA) catabolism. For this reason, GABA concentrations in the central nervous system increase and thereby induce a decrease in brain activity [14]. It functions as a GABA agonist, and its mechanism of action is similar to that of benzodiazepines [15].

Valerian can be marketed in multiple dosage forms, including dry extract, tincture, and dry herbal substance. The traditional way of consuming it is the direct ingestion of the root pulverized in boiling water [16,17]. It has an easy administrative method and, in addition, its low toxicity has contributed to an exponential increase in its use in therapeutics for the approach of different pathologies, including insomnia, mild anxiety.

Currently, there are no standardized doses when they are marketed as infusions or food supplements, although the European Medicines Agency (EMA) has established that the effective doses are 2–3 g in infusion [18]. Also, for optimal treatment, it should be administered for at least two weeks.

Although medicinal plants can be used at certain doses for the treatment of certain pathologies, it is known that most medicinal plants used by the population are a source of toxic metals and minerals [19,20,21]. Therefore, quality controls are important for a safe intake [17,22,23,24,25].

The contamination of certain plants with toxic metals, due to environmental pollution, is a documented fact [26,27,28,29,30], especially due to the increase in areas of cultivation close to sources of anthropogenic contamination and by the ability of some plants to selectively accumulate certain elements. For this reason, the control of the content of toxic metals, especially lead (Pb) and cadmium (Cd), has been established as one of the quality controls by international institutions [31].

For example, Cd is a highly nephrotoxic toxic metal; it is able to accumulate in the kidneys of animals and produce kidney failure, but with prolonged exposure it can also lead to osteoporosis, diabetes, cardiovascular diseases and cancer [32]. Pb is able to interact with the thiol groups of proteins, inhibiting certain enzymes and interfering with the homeostasis of magnesium, calcium and zinc.

It also increases oxidative stress, interacting with the proteins of the liver, kidney and red blood cells, which will cause toxicity in these tissues, and it interferes with bone mineralization, the cardiovascular system, the reproductive system and the neurological system.

In addition to their well-documented systemic effects, cadmium (Cd) and lead (Pb) also exert profound neurotoxic effects, particularly in the context of chronic exposure [10,33]. Both metals can cross the blood–brain barrier and accumulate in brain tissue, where they induce oxidative stress, neuroinflammation, and neuronal apoptosis, mechanisms directly implicated in the pathogenesis of neurodegenerative diseases such as Alzheimer’s and Parkinson’s disease [34].

Cadmium accumulates in the hippocampus and cortex, disrupting redox signaling and synaptic function [35]. Lead similarly enters the brain via calcium-transport pathways and interferes with neurotransmission, ionic balance, and cellular metabolism [34]. These neurotoxic mechanisms are of particular concern in populations exposed through environmental contamination [36].

Aluminum (Al) is another highly toxic metal, especially affecting the nervous and hematopoietic system, but it also interferes with bone mineralization and is carcinogenic, all due to its ability to inhibit a multitude of physiological enzymes [32].

Regarding the rest of the metals and elements studied, they are considered either as trace elements or macroelements, as they are all considered to be of nutritional interest. Therefore, their determination is important to define the metal profile of a substance. Trace elements such as chromium (Cr), copper (Cu), lithium (Li), nickel (Ni), strontium (Sr), molybdenum (Mo), zinc (Zn), cobalt (Co), iron (Fe), boron (B), manganese (Mn), vanadium (V) and barium (Ba), these are hormetic, so they can be potentially toxic [37]. The macroelements are potassium (K), sodium (Na), calcium (Ca) and magnesium (Mg), and are necessary in human metabolism.

Table 1 shows the recommendations or dietary exposure limits established by the European Food Safety Authority (EFSA) for each studied element. If the EFSA has not established any reference value, the value of another recognized health institution that does have one shall be used.

The World Health Organization (WHO) has set the maximum recommended concentration for lead in medicinal plants at 10 mg/kg and for cadmium at 0.3 mg/kg [31]. However, the European Pharmacopoeia sets general limits for medicinal plants of 5 mg/kg for lead and 0.1 mg/kg for cadmium, unless other limits are specified in the individual monographs [47]. Meanwhile, the Royal Spanish Pharmacopoeia does not specify general limits but rather refers to the limits established in each monograph [48], and neither the European Pharmacopoeia nor the Royal Spanish Pharmacopoeia set specific limits for toxic metals in the monograph of the *Valeriana officinalis* L. root.

At the legislative level, Commission Regulation (EU) 2023/915 [49] established in general terms for food supplements the parametric values of 3 mg/kg for lead and 1 mg/kg for cadmium [49]. The medicinal plants placed on the market are classified in this category form according to European regulations [50].

Furthermore, it should be noted that underground organs are one of the main sources of nutrients and metals absorbed by plants [51], so they may be among the organs where contaminants accumulate the most. Many plants have a special tendency to accumulate metals and minerals in their roots, and certain species may be used for the remediation of contaminated soils [52,53,54].

For all the above, it is of interest to research the composition of metals, those of toxicological interest (Pb, Cd and Al) and those of nutritional interest (Cr, Cu, Li, Ni, Sr, Mo, Zn, Co, Fe, B, Mn, V, Ba, Ca, Mg, K and Na), in the marketed medicinal plant forms most used by the population. Especially when the organs of interest are the roots and when the recommended intake is multiple daily doses over a long term, as valerian.

The composition of certain metals in valerian root has been the subject of few previous studies [55,56,57,58], but in no case has the metal profile of this plant organ been determined for consumption.

In this study, we have determined the metal profile of the medicinal plant *Valeriana officinalis* L., commercialized as an herbal plant or as a dietary supplement, to assess whether it meets the quality criteria established for its commercialization. The toxic risk assessment of consuming this medicinal plant, in any of its forms, at the recommended doses for its use in phytotherapy, has also been evaluated.

## 2. Materials and Methods

### 2.1. Samples

A total of 23 samples of different commercialized brands of *Valeriana officinalis* L. were selected in various forms (fragmented, crushed and pulverized) as a herbal plant or as a dietary supplement. The criterion for selection was that the sample forms must be a herbal substance of valerian root in any of its forms, discarding the forms of dry extract and tinctures. The samples included represent all those that could be obtained through commercial channels in our environment and that met the selection criteria. All samples were acquired in February 2025 from stores on the island of Tenerife and stored under appropriate conditions (at room temperature in a cool, dry place, protected from sunlight). Table 2 shows a summary of the types of valerian root forms selected for this study.

It should be noted that in the case of crushed valerian root and the one presented in infusers, only one sample, the one purchased in a pharmacy, was composed of 100% crushed valerian root. The rest of the samples, although they were marketed claiming to be “Valerian”, they contained crushed valerian root in variable proportions, ranging from 15 to 80%. The remaining composition being other plant drugs with a similar activity such as passionflower, lemon balm or lime blossom among others, or in many cases other plant drugs added to mask the organoleptic properties of the valerian root and make them more pleasant to the consumer, as is the case of aniseed, fennel, mint, licorice or apple.

However, this was not taken into account for the purposes of the study, since these were forms marketed as “prepared valerian infusers” and it was assumed that the general population would consume them as such.

As all samples were acquired in European territory, they had CE markings, so they had adequate quality control carried out by the manufacturer, who certifies the identification of the species of interest.

### 2.2. Sample Treatment and Analytical Method

#### 2.2.1. Sample Treatment

Two aliquots of 10 g were taken after homogenization of the samples in porcelain capsules (Staatlich, Berlin, Germany), which were dried in the oven (Nabertherm, Germany) at 80 °C for 24 h. They were then subjected to acid digestion to destroy the organic matter. To do this, a sufficient amount of 65% HNO_3_ (Sigma Aldrich, Taufkirchen, Germany) was added to each porcelain crucible with each aliquot, and it was placed on the heating plate (Nabertherm, Lilienthal, Germany) until evaporation, and finally incinerated in a muffle furnace (Nabertherm, Lilienthal, Germany).

Due to the complexity of the sample, a first temperature-time program of 400 °C for 24 h was necessary, with a progressive increase in temperature of 16 °C/h, and after that, a second acid digestion following the same procedure, a second temperature-time program of 400 °C for 12 h, with a progressive increase in temperature of 32 °C/h.

Once the ashes were obtained, they were dissolved in a 1.5% HNO_3_ solution (Sigma Aldrich, Taufkirchen, Germany) up to 0.025 L, which were stored in appropriate polyethylene containers for subsequent analysis [37,59,60]. It should be noted that the ashes never became completely white due to the high pigment content in these types of samples, and filtration was necessary to dissolve them because of the large amount of insoluble ashes that were formed due to the high silicon content [61,62,63,64], which is contemplated in the respective monographs of the European Pharmacopoeia and the Royal Spanish Pharmacopoeia [47,48].

#### 2.2.2. Instrumentation and Method Validation

The metal content was determined by ICP-OES (inductively coupled plasma optical emission spectrometry) [65,66]. The ICP model was iCAP 6300 Duo Thermo Scientific (Waltham, MA, USA) equipped with an automatic sampler (CETAX model ASX-520, Omaha, NE, USA) and with a CID86 chip (Charge Injection Device, Thermo Fisher Scientific, Waltham, MA, USA) that provided the user with a possible choice of wavelengths from 166 to 847 nm. This model had exceptional analytical versatility, optimizing purge-gas consumption and significantly improving the signal-to-noise ratio over the entire concentration range. Its stability and low noise level made it a suitable model for this type of analysis.

The instrumental conditions of the ICP-OES were as follows: approximate flow power rate, 1150 W; gas flow (nebulizer and auxiliary), 0.5 L/min; sample injection to flow pump, 45 rpm; stabilization time, 0 s. Liquid argon (99.999% purity, Air Liquide, Tenerife, Spain) was employed in the ICP-OES metal analysis.

Analytical calibration curves were prepared daily prior to sample measurement. The calibration curves were prepared from a multi-element stock solution Multi-Element Std. SCP28AES (SCP Science, Baie-D’Urfe, QC, Canada) of 100 mg/L V, Mn, Fe, Cu, Zn, Cr, Mo, Co, B, Ba, Li, Sr, Ni, Si, Al, Pb and Cd (Merck, Darmstadt, Germany) and a stock solution IV-STOCK-2 of 10,000 μg/mL Ca, Na, Mg, K (Inorganic Ventures, Christianburg, VA, USA). Batch standards other than those used for sample addition were used to prepare calibration curves in order to maintain the accuracy and precision parameters of the method. Calibration curves were prepared using 1.5% nitric acid (HNO_3_) (Merck, Darmstadt, Germany).

Quality controls were conducted based on the recovery percentage study obtained with the reference material under reproducible conditions. This was done to verify the accuracy of the analytical procedure. The certified reference materials used were kept in a desiccator under controlled conditions of temperature and humidity. These were as follows: SRM Oyster Tissue 1566b, SRM 1573a Tomato Leaves and SRM 1515 Apple Leaves (Gaithersburg, MD, USA).

The method has been validated in line with the parameters set out in the European Commission Regulation (EC) No 333/2007 of 28 March 2007, which lays down the methods of sampling and analysis for official control. Monitoring and control of lead, cadmium, mercury, inorganic tin, 3-MCPD and benzo(a)pyrene levels in foodstuffs (EC, 2007, 2011, 2016) [67,68,69].

The validation parameters that have been verified are as follows:

1. Specificity: The method has been verified to be free of spectral interferences for each of the metals studied.
2. Precision: Based on reproducibility, the method has been checked for each of the metals with a HorRatr value of less than 2.
3. Accuracy, based on recovery, has been demonstrated by recoveries of between 98.7 and 100.6% across all samples, with the lowest recoveries observed in the enriched samples at the method’s quantification limit. It is important to note that no correction or recovery factor should be applied, as the use of reference materials has demonstrated that the certified reference concentration is reached, and the concentration added was found in the case of the enriched samples.

Furthermore, a statistical analysis was conducted to confirm the absence of significant differences between the measured concentrations and the certified values. The obtained concentrations exhibited a high degree of correlation with the certified concentrations, with relative standard deviation (RSD) values below 25%.

In accordance with the IUPAC (1995) [70] guidelines, the detection and quantification limits (Table 3) have been calculated under reproducibility conditions and are three and ten times the standard deviation (SD) resulting from the analysis of 15 targets.

The aforementioned parameters have been verified through the measurement of 10 samples with concentrations approaching the limits of quantification for each metal, as well as the assessment of 10 samples of reference materials under reproducibility conditions. These latter samples serve as quality controls for the methodology.

Table 3 shows the operating parameters of the ICP-OES, including emission wavelength, limit of detection (LOD) and limit of quantification (LOQ).

After obtaining the concentration in the different forms of valerian root, it was compared with the established safety and quality parameters. Given the many different parameters established by the different laws, monographs and recommendations, the most demanding one for each case was taken as the reference. All marketed forms of valerian root must comply with the maximum content parameter of 3 mg/kg of lead according to Regulation (EU) 2023/915 for food supplements, and with the maximum content parameter of 0.1 mg/kg of cadmium according to the European Pharmacopoeia [47].

### 2.3. Statistical Analysis

A statistical analysis was conducted to ascertain whether there were any notable discrepancies in the metal concentrations present in the various commercial forms of valerian root (fragmented in a bag, crushed in infusers and pulverized in capsules). A *p*-value of less than 0.05 [71] was considered to indicate a statistically significant difference. The statistical analysis of the samples was performed using the IBM Statistics SPSS Program (version 24.0, IBM Company, Armonk, NY, USA).

The Kolmogorov–Smirnov and Shapiro–Wilk tests were applied to verify the normality of the data obtained and the Levene test was performed to evaluate the homogeneity of the variances [72,73]. When there was normality of the data, parametric tests were conducted (ANOVA and post hoc test), and in the case of no normality, non-parametric tests were performed (Kruskal-Wallis H test and Mann–Whitney U test) [60,72].

### 2.4. Risk Assessment

Once the metal profile of the valerian root samples was determined, the EDI of the different elements derived from their consumption was calculated, especially those of toxicological interest (Cd, Pb and Al), in the doses and recommended dosage to obtain the therapeutic effect. Therefore, two grams was taken as a mean average therapeutic dose by European Medicines Agency, (2015) [18], regardless of its forms and the manufacturer’s recommendations, with a variable dosage of a single dose per day or three daily doses depending on whether a specific anxiolytic effect (for sleeping) or permanent anxiolytic effect (for states of stress) is intended, respectively.

$$EDI=Concentration\;detected\;\left(\frac{mg}{kg}\right)\times Therapeutic\;average\;dose\left(\frac{g}{dose}\right)\times Posology\;\left(\frac{dose}{day}\right)$$

Once the EDIs were obtained, they were compared with the limits and/or recommendations established by the main international health organizations (Table 1).

## 3. Results and Discussion

Although it was not an objective of the present study, it is worth mentioning that during the processing of the samples the percentage of humidity of the samples could be checked, a test that is usually carried out in all plant drugs to avoid the proliferation of microorganisms, the limit being usually set between 10 and 14% [74,75,76].

In the samples analyzed in this study, none exceeded 10% humidity, which satisfies the quality test for loss of weight by drying described in the specific monographs of the European Pharmacopoeia and the Royal Spanish Pharmacopoeia, which establishes that 12.0% should not be exceeded [47,48].

Table 4 shows the average concentration (mg/kg) of the elements analyzed in the samples whose commercial form is fragmented root, which were presented in packaged bags or were purchased in loose format in different markets.

Table 5 shows the mean average concentration (mg/kg) of the elements analyzed in the samples whose commercial forms is crushed root, which were presented in infusers. The percentage of valerian root that each of them contained is shown below each brand, because as mentioned above, although they are marketed as “Valerian”, their composition is a complex mixture of medicinal plants to make them more acceptable to the consumer. This information is not taken into account in the present study when attributing the possible source of the metals.

Table 6 shows the mean average concentration (mg/kg) of the metals analyzed in the samples whose commercial forms is pulverized root, which were presented in capsules or pills. The gelatin cover was not considered in the determination.

Table 7 shows the mean average concentration (mg/kg) of each metal in each type of valerian root form. The fragmented valerian root is called “Bag”, the crushed one is called “Infusers”, and the pulverized one is “pills”.

The results obtained show that no forms exceed the safety parameters for the concentration of Pb, with the concentration of all samples being below 0.1 mg/kg, which is well below the 3 mg/kg which is the most demanding established limit.

Regarding the concentration of Cd, the mean average concentration of the studied forms does not exceed the safety parameter of 0.1 mg/kg, although in this case the one presented in a bag (fragmented) is close to this limit. However, it should be noted that, if we do the analysis by sample, there are brands that exceed this limit in all forms. In the bag forms (fragmented), there are two brands that exceed this value: Pinisan (0.11 mg/kg) and one of the market samples (0.23 mg/kg). In the infuser forms (crushed), there is one brand that exceeds this value: EnRelax (0.13 mg/kg). And in pill forms (pulverized), there are three brands exceeding this value: EnRelax (0.12 mg/kg), Kneipp (0.15 mg/kg) and Milvus (0.16 mg/kg).

The statistical analysis determined that, among the metals following a normal distribution (Al, Zn, Cu, Mg and Na), the only one that had significant differences between the different groups of forms is Na. In the case of the other elements, which did not follow a normal distribution (Cd, Pb, Cr, Li, Ni, Sr, Mo, Co, Fe, B, Mn, V, Ba, K and Ca), the ones that present significant differences between the different groups of forms are Co, Fe, B, Mn, V, Ca, Sr, Li and K.

The other elements did not have significant differences in the concentration for the different forms of valerian. It should be noted that, for metals with significant differences between the form groups, the highest concentrations usually corresponded to the bag or loose format (fragmented) forms group, except for Na, B, Ca, and Sr, in which case the highest concentration was found in the group of forms in infusers (crushed).

Comparable Cd, Cu and Cr levels in valerian roots (Cd (0.03 mg/kg), Cu (2.71 mg/kg) and Cr (2.08 mg/kg)) have been reported by Martín-Domingo (2017) [56], who attributed metal accumulation to soil characteristics and cultivation practices, supporting the trends observed in the present study. in samples acquired in Spain, although the concentrations of Pb (0.98 mg/kg), Fe (376 mg/kg), Zn (33.8 mg/kg) and Mn (38.8 mg/kg) found in the present study are lower. Furthermore, they were generally much lower than those found by Arce et al. (2005) [55] in samples acquired in Argentina (Cu 4.97 ± 0.15 mg/kg; Mn 83.47 ± 2.5 mg/kg; Fe 2293 ± 45.86 mg/kg; Zn 27.3 ± 0.82 mg/kg; Ni 4.5 ± 0.18 mg/kg; Pb 17.04 ± 0.68 mk/Kg), except for the Cd content (0.0125 ± 0.0006 mg/kg), which is an indication of the importance of the origin of the samples.

Finally, studies conducted by Jurowski et al. [57,58] on products obtained from Polish pharmacies have found similar concentrations of Cu (0.16–0.23 mg/L), but much lower concentrations of Mn (0.11–0.76 mg/L), Zn (0.22–0.48 mg/L), Pb (0.55–1.85 μg/L), and Cd (0.051–0.27 μg/L). It is important to note that the products analyzed in this latest study are made from plant organ extracts and are marketed as herbal medicinal products.

If we compare the data above with the concentrations found in other medicinal plants, it can be observed that the concentrations found in the present study are overall lower than those found in African medicinal plants, such as mint, ginkgo, centella asiatica, passionflower, senna, aloe, ginseng, among others, except those found for Cd, which tend to be similar or higher [56,77,78,79].

These concentrations were also found to be lower than those found in certain algae, except for Cr and Sr, although they were overall higher than those found in other food supplements such as proteins [59,60,80,81].

Table 8 shows the EDI of each element that would be ingested with the consumption of a single daily therapeutic dose if consumed to help sleep and Table 9 shows the EDI of each element with the consumption of three daily therapeutic doses if it were consumed to treat mental stress.

On the basis of the IDEs obtained, the amount of elements provided by the intake of valerian when used in doses recommended in phytotherapy, does not pose a toxicological risk, as all exposures are well below the intake limit values (Table 1) set by major international health organizations even at the highest doses. However, it is advisable to take them into account as a possible source of metals in the overall intake. Furthermore, it should be noted that the consumption of higher doses, with greater frequency, or over the long term, should be evaluated the risk assessment.

Furthermore, when considering the sources of these elements, the contribution of different sources of dietary exposure must also be considered in order to assess whether or not there is a toxicological risk. Within this framework, the percentage contribution to the toxicological reference values for Al and Cd has been calculated for an adult population (70 kg), as these are the intrinsically toxic elements among those analyzed that do not follow the MOE approach (Table 10).

In all cases, the percentages are less than 1%, so it can be guaranteed that, regardless of the forms format, the matrix analyzed does not pose a risk of dietary overexposure to these metals.

## 4. Conclusions

The analysis demonstrated that in the case of Cd, six samples exceeded the most stringent parameter, which is set at 0.1 mg/kg. The samples that exceeded the indicated Cd content were as follows: the results of the quality controls revealed that Pinisan in bags (0.11 mg/kg), EnRelax in capsules (0.12 mg/kg), EnRelax in infusers (0.13 mg/kg), Kneipp in tablets (0.15 mg/kg), Milvus in capsules (0.16 mg/kg) and the market bulk sample (0.23 mg/kg) all contained concentrations of toxic metals (Pb and Cd) that exceeded the most demanding parameter for Pb (set at 3 mg/kg) and the most stringent parameter for Cd (set at 0.1 mg/kg). It is recommended that the quality controls be increased, particularly in regard to Cd.

It should be noted that the study may have certain limitations, including the following: it is challenging to determine the origin of the marketed medicinal plants, as it is not a legal requirement to specify it; in herbal dietary supplements intended to improve a disease condition, it is common for them to contain a mixture of plants, even when the name of the supplement seems to indicate that it has only one.

Regarding the metal content, it can be concluded that there is no toxicological risk associated with the intake of any of the metals based on the consumption scenario studied for the therapeutic intake of valerian root, irrespective of the form in which it is consumed, including the highest dose. Furthermore, it should be noted that, based on the results obtained, it is necessary to conduct more exhaustive studies regarding the concentration of toxic metals in medicinal plants, as well as to improve and update the legislation concerning the presence of these metals in such plants.

## Figures and Tables

**Table 1:** Limit or recommended intake values for adults of the analyzed metals, set by the main health organizations.

Element	Parameter	Value	Reference
Cd	TWI	2.5 μg/kg bw/week	[38]
Pb	BMDL	0.50 μg/kg bw/day ^1^ 0.63 μg/kg bw/day ^2^ 1.50 μg/kg bw/day ^3^	[39]
Al	TWI	1 mg/kg bw/week	[38]
Cr	TDI	0.3 mg/kg bw/day	[40]
Cu	AI UL	♂ 1.6 mg/day–♀ 1.5 mg/day 5 mg/day	[41] [42]
Li	p-RfD	2 μg/kg bw/day	[43]
Ni	TDI	13 μg/kg bw/day	[44]
Sr	TDI	0.13 mg/kg bw/day	[31]
Mo	AI UL	65 μg/day 0.6 mg/day	[41] [42]
Zn	AI UL	9.4–16.3 mg/day ^4^ 25 mg/day	[40] [42]
Co	SLI ^5^	600 μg/day ^5^	[45]
Fe	AI	♂ 11 mg/day–♀ 16 mg/day	[41]
B	UL	10 mg/day	[42]
Mn	AI SLI	3 mg/day 8 mg/day	[41] [42]
V	RfD	7 μg/kg bw/day	[43]
Ba	TDI	0.2 mg/kg bw/day	[43]
K	AI	3500 mg/day	[41]
Na	AI	2000 mg/day	[46]
Ca	AI UL	950 mg/day 2500 mg/day	[41] [42]
Mg	AI UL	♂ 350 mg/day–♀ 300 mg/day 250 mg/day ^6^	[41] [42]

TWI, tolerable weekly intake; BMDL, benchmark dose level; TDI, tolerable daily intake; AI, adequate intake; UL, tolerable upper intake level; p-RfD, provisional subchronic and chronic reference dose; RfD, oral reference dose; SLI, safe level of intake; bw, body weight. ^1^ For neurotoxicity. ^2^ For nephrotoxicity. ^3^ For cardiovascular effects. ^4^ Depending on the level of phytate intake. ^5^ Although an official parameter has not been established, it has been estimated by EFSA as a safe acceptable daily oral intake, with a minimum level of risk. ^6^ Provided by nutritional supplements, without taking into account the Mg normally present in foods and drinks.

**Table 2:** Types of valerian root forms selected for this study.

	Fragmented	Crushed	Pulverized
Number of samples	8	7	8
Sample breakdown	5 samples in “bag” format 3 samples in “bulk” format	7 samples in filters for infusion	7 samples formulated in capsules 1 sample formulated in dragees
Place of acquisition	Herbalists and markets	Herbalists, supermarkets and pharmacies	Supermarkets and pharmacies

**Table 3:** Operational parameters for inductively coupled plasma optical emission spectrometry (ICP-OES).

Metal	Wavelength (nm)	LOD (mg/L)	LOQ (mg/L)
Cd	214.4	0.0007	0.002
Pb	220.3	0.0009	0.003
Al	167.0	0.005	0.015
Cr	267.7	0.001	0.005
Cu	324.7	0.003	0.011
Li	670.7	0.013	0.031
Ni	221.6	0.0009	0.003
Sr	407.7	0.003	0.011
Mo	202.0	0.0016	0.005
Zn	213.8	0.0027	0.009
Co	228.6	0.001	0.005
Fe	238.2	0.004	0.013
B	249.6	0.008	0.027
Mn	257.6	0.0008	0.003
V	292.4	0.0014	0.004
Ba	455.4	0.0006	0.002
K	766.4	1.764	5.883
Na	818.3	2.221	7.404
Ca	315.8	1.629	5.432
Mg	383.8	1.580	5.268

**Table 4:** Mean concentrations of each element (mg/kg) in valerian root samples in fragmented root forms (in bags and in loose format).

Metal	Commercial Brands							
	Plameca	Naturcid	Pinisan	Soria Natural	Herbofarma	Market 1	Market 2	Market 3
Al	49.70	20.26	65.15	11.53	41.66	54.17	35.18	14.54
Mo	0.020	0.020	0.014	0.012	0.029	0.013	0.017	0.019
Zn	21.43	13.00	13.62	5.22	6.95	8.54	10.33	4.69
Cd	0.10	0.10	0.11	0.017	0.032	0.059	0.26	0.037
Pb	N.D.	0.025	0.10	0.0013	0.010	0.064	0.021	0.0037
Ni	0.21	1.09	0.23	0.12	0.11	0.16	1.01	0.66
Co	0.011	0.11	0.034	0.025	0.033	0.025	0.19	0.11
Fe	40.94	14.88	109.09	6.49	25.13	48.72	40.76	12.28
B	3.81	3.26	2.22	2.03	3.50	2.31	2.29	1.96
Mn	3.49	26.63	8.51	2.87	3.74	5.20	18.57	9.22
Cr	3.54	0.82	1.60	1.86	2.58	3.15	4.28	5.66
V	0.15	0.075	0.19	0.042	0.13	0.12	0.12	0.078
Ca	1881.74	941.54	1991.70	502.20	966.11	1439.95	984.65	567.93
Cu	1.12	1.11	1.17	0.37	0.56	0.64	0.90	0.47
Mg	3710.83	3136.58	2252.13	2063.43	2910.60	2222.40	3316.14	2635.71
Sr	4.21	2.49	4.05	1.35	3.14	3.33	1.87	1.23
Ba	2.24	2.94	2.89	0.90	1.73	2.82	1.73	1.21
Li	0.47	0.64	0.64	0.84	0.51	0.61	0.39	0.39
K	12,291.92	10,774.96	7969.79	9238.28	10,501.12	9016.44	10,285.79	9692.39
Na	401.77	303.22	603.35	540.26	888.41	405.82	231.33	169.42

N.D.—Not detectable.

**Table 5:** Mean concentrations of each element (mg/kg) in valerian root samples in crushed root forms (in infusers).

Metal	Commercial Brands *						
	Naturalista (80%)	La Pirenaica (100%)	Artemis (40%)	EnRelax (15%)	Pompadour (20%)	Hornimans (50%)	Salus Floradix (30%)
Al	33.96	11.22	6.91	2.25	2.99	42.07	28.40
Mo	0.021	0.059	0.12	0.024	0.017	0.0088	0.051
Zn	5.98	1.14	3.66	6.00	0.70	4.86	12.10
Cd	0.024	0.010	0.017	0.13	0.0037	0.028	0.029
Pb	N.D.	N.D.	N.D.	N.D.	N.D.	0.016	0.096
Ni	0.16	0.17	0.28	0.21	0.080	0.25	1.54
Co	0.0085	0.0088	0.029	0.0037	N.D.	0.0088	0.46
Fe	11.76	2.61	6.63	2.07	2.61	17.96	10.39
B	5.37	3.99	7.70	4.95	1.67	7.19	3.25
Mn	1.36	0.39	0.98	0.45	0.32	2.86	15.0
Cr	2.31	4.46	0.17	0.30	0.35	1.13	4.58
V	0.055	0.029	0.029	0.0062	0.0085	0.063	0.52
Ca	1263.16	1545.07	3475.10	5579.74	3975.87	1179.83	1308.28
Cu	0.66	0.14	0.76	0.15	0.079	0.58	1.33
Mg	2464.43	2075.93	2401.55	1908.23	997.20	2138.76	2303.15
Sr	3.46	3.77	11.40	29.92	18.82	3.97	3.30
Ba	1.52	2.23	2.41	12.85	4.60	2.64	1.57
Li	0.31	0.21	0.34	0.16	0.17	0.41	0.77
K	9203.43	10,646.76	10,447.74	9765.40	9300.94	6574.60	9468.05
Na	307.08	413.34	556.10	682.63	279.70	1415.41	674.34

N.D.—Not detectable. * Although all the forms are marketed as “valerian”, most are mixed with other plant drugs. In each case, the proportion of valerian it contains is indicated, although for practical purposes, this is not taken into account.

**Table 6:** Mean concentrations of each element (mg/kg) in valerian root samples in pulverized root forms (in capsules or pills).

Metal	Commercial Brands *							
	Arkocapsulas	Deliplus	EnRelax	Milvus	Ebers	Vive+	Vitalissima	Kneipp
Al	4.46	28.21	1.53	22.70	30.28	39.10	12.87	23.83
Mo	0.060	0.027	0.022	0.023	0.0050	0.018	0.010	0.018
Zn	4.24	1.40	7.28	14.92	8.96	3.80	1.96	13.58
Cd	0.026	0.0081	0.12	0.16	0.10	0.025	0.010	0.15
Pb	N.D.	0.014	N.D.	0.037	0.014	N.D.	0.0030	0.076
Ni	0.18	0.18	0.17	0.60	0.26	0.30	0.24	0.18
Co	0.0060	0.0069	0.0024	0.0090	0.0075	0.012	0.0045	0.017
Fe	5.21	21.09	1.49	16.78	15.08	16.19	9.30	34.63
B	8.64	1.15	2.38	2.70	1.87	2.40	2.27	1.17
Mn	1.33	0.84	0.16	1.86	3.30	1.67	0.67	1.58
Cr	0.10	1.56	0.80	6.06	0.46	1.22	0.74	0.24
V	0.023	0.046	0.0036	0.035	0.040	0.077	0.040	0.16
Ca	4533.05	778.67	4671.95	1212.37	796.94	1101.70	747.58	1379.29
Cu	0.26	0.14	0.11	0.47	0.51	0.78	0.30	1.34
Mg	1778.64	3433.55	992.53	2624.39	2108.25	3268.80	2893.04	3482.78
Sr	20.16	1.71	17.93	3.51	2.28	2.81	2.18	3.50
Ba	5.26	0.89	9.45	2.30	2.39	1.51	1.14	1.93
Li	0.23	0.25	0.32	0.16	0.28	0.28	0.13	0.14
K	10,162.42	7848.24	9705.95	8104.28	6002.02	6924.37	5301.06	3708.38
Na	766.88	245.49	282.83	287.90	334.59	335.05	203.49	312.67

N.D.—Not detectable. * All selected brands are presented in capsule form. The Kneipp brand is the only one that comes in pill form.

**Table 7:** Mean and standard deviation of the concentrations of each element (mg/kg) in the valerian root samples in the groups of valerian root forms: Bag (fragmented), infusers (crushed) and pills (pulverized).

Metal	Bag	Infusers	Pills
Al	36.52 ± 19.66	18.26 ± 16.25	20.37 ± 13.05
Mo	0.019 ± 0.006	0.042 ± 0.038	0.022 ± 0.016
Zn	10.47 ± 5.52	4.92 ± 3.82	7.02 ± 5.14
Cd	0.091 ± 0.079	0.034 ± 0.042	0.075 ± 0.064
Pb	0.028 ± 0.036	0.012 ± 0.038	0.017 ± 0.028
Ni	0.45 ± 0.41	0.38 ± 0.51	0.26 ± 0.14
Co *	0.067 ± 0.062	0.074 ± 0.17	0.008 ± 0.005
Fe *	37.30 ± 32.80	7.72 ± 5.97	14.97 ± 10.27
B *	2.67 ± 0.73	4.87 ± 2.13	2.82 ± 2.42
Mn *	9.78 ± 8.52	3.05 ± 5.34	1.42 ± 0.95
Cr	2.94 ± 1.57	1.90 ± 1.93	1.40 ± 1.94
V *	0.12 ± 0.048	0.10 ± 0.18	0.053 ± 0.047
Ca *	1159.48 ± 559.38	2618.15 ± 1737.85	1902.69 ± 1681.84
Cu	0.79 ± 0.32	0.53 ± 0.45	0.49 ± 0.41
Mg	2780.98 ± 588.21	2041.32 ± 499.32	2572.75 ± 886.59
Sr *	2.71 ± 1.16	10.66 ± 10.30	6.76 ± 7.63
Ba	2.06 ± 0.79	3.98 ± 4.05	3.11 ± 2.90
Li *	0.56 ± 0.15	0.34 ± 0.21	0.22 ± 0.074
K *	9971.34 ± 1306.23	9343.84 ± 1341.61	7219.59 ± 2190.86
Na *	442.95 ± 231.71	618.37 ± 387.31	346.11 ± 175.78

* The indicated metals show statistically significant differences in their concentrations between the different groups of forms.

**Table 8:** Estimated Daily Intake (EDI) of each element (mg) with a single dose for each of the groups of valerian root forms: Bag (fragmented), infusers (crushed) and pills (pulverized).

Metal	Bag	Infusers	Pills
Al	0.073	0.037	0.041
Mo	3.7 × 10^−5^	8.5 × 10^−5^	4.5 × 10^−5^
Zn	0.021	0.010	0.014
Cd	1.8 × 10^−4^	6.8 × 10^−5^	1.5 × 10^−4^
Pb	5.6 × 10^−5^	2.3 × 10^−5^	3.4 × 10^−5^
Ni	9.0 × 10^−4^	7.7 × 10^−4^	5.3 × 10^−4^
Co	1.3 × 10^−4^	1.5 × 10^−4^	1.6 × 10^−5^
Fe	0.075	0.015	0.030
B	5.3 × 10^−3^	9.7 × 10^−3^	5.6 × 10^−3^
Mn	0.020	6.1 × 10^−3^	2.9 × 10^−3^
Cr	5.9 × 10^−3^	3.8 × 10^−3^	2.8 × 10^−3^
V	2.3 × 10^−4^	2.0 × 10^−4^	1.1 × 10^−4^
Ca	2.32	5.24	3.80
Cu	1.6 × 10^−3^	1.1 × 10^−3^	9.8 × 10^−4^
Mg	5.56	4.08	5.14
Sr	5.4 × 10^−3^	0.021	0.014
Ba	4.1 × 10^−3^	0.8 × 10^−3^	6.2 × 10^−3^
Li	1.1 × 10^−3^	6.8 × 10^−4^	4.5 × 10^−4^
K	19.94	18.69	14.44
Na	0.89	1.24	0.69

**Table 9:** Estimated Daily Intake of each element (mg) with three daily doses for each of the groups of valerian root forms: Bag (fragmented), infusers (crushed) and pills (pulverized).

Metal	Bag	Infusers	Pills
Al	0.22	0.11	0.12
Mo	1.1 × 10^−4^	2.5 × 10^−4^	1.3 × 10^−4^
Zn	0.063	0.030	0.042
Cd	5.4 × 10^−4^	2.0 × 10^−4^	4.5 × 10^−4^
Pb	1.7 × 10^−4^	7.0 × 10^−5^	1.0 × 10^−4^
Ni	2.7 × 10^−3^	2.3 × 10^−3^	1.6 × 10^−3^
Co	4.0 × 10^−4^	4.4 × 10^−4^	4.9 × 10^−5^
Fe	0.22	0.046	0.090
B	0.016	0.029	0.017
Mn	0.059	0.018	8.6 × 10^−3^
Cr	0.018	0.011	8.4 × 10^−3^
V	6.9 × 10^−4^	6.0 × 10^−4^	3.2 × 10^−4^
Ca	6.96	15.71	11.42
Cu	4.8 × 10^−3^	3.2 × 10^−3^	2.9 × 10^−3^
Mg	16.69	12.25	15.44
Sr	0.016	0.064	0.041
Ba	0.012	0.024	0.019
Li	3.4 × 10^−3^	2.0 × 10^−3^	1.3 × 10^−3^
K	59.83	56.06	43.32
Na	2.66	3.71	2.08

**Table 10:** Percentages of contribution to the TWIs of Cd and Al for the adult population.

Metal	Bag	Infusers	Pills
Al	0.31%	0.16%	0.17%
Cd	0.33%	0.11%	0.26%

## Data Availability

The original contributions presented in the study are included in the article. Further inquiries can be directed to the corresponding author.
